# Editorial: Community series in the role of angiogenesis and immune response in tumor microenvironment of solid tumor, volume III

**DOI:** 10.3389/fimmu.2024.1495465

**Published:** 2024-10-14

**Authors:** Fang Zheng, Zilong Chen, Wenqing Jia, Ren Zhao

**Affiliations:** ^1^ Department of General Surgery, Ruijin Hospital, Shanghai Jiao Tong University School of Medicine, Shanghai, China; ^2^ Shanghai Institute of Digestive Surgery, Ruijin Hospital, Shanghai Jiao Tong University School of Medicine, Shanghai, China

**Keywords:** angiogenesis, immune response, tumor microenvironment, immunotherapy, vascular endothelial growth factor

Tumorogenesis is characterized by the unregulated proliferation of cells, which is the consequence of dysregulated cell division mechanisms. To ensure rapid multiplication and invasion, a tumor tends to create a unique tumor microenvironment (TME) benefiting its own progression. As an abnormal component in the body, figuring out how to escape from the supervision and attack of the immune system is key to tumor survival. Angiogenesis, which is mainly triggered by the stimulation of vascular endothelial growth factor (VEGF), is one of its solutions. Characterized by hypoxia, most types of tumor cells significantly upregulate VEGF, which stimulates the active construction of tumor neovascularization (1–3). However, the newly formed tumor blood vessels show a series of abnormalities, including increased luminal resistance, increased endothelial permeability, and limited blood flow caused by defects in structure (4). The features above are beneficial to tumor growth and metastasis as well as immune evasion, inducing resistance to immunotherapy. It was reported that anti-angiogenesis therapy has been seen as a promising strategy to enhance tumor immune response, which contributes to improving immunosuppressive TME and aids the immune system’s anti-tumor effects (5–7).

The relation between angiogenesis and immune response was revealed at the gene and transcriptomic level. Kurmyshkina et al. have focused on the difference in transcriptomic landscapes between early-stage invasive cervical carcinoma (CeCa) and cervical intraepithelial neoplastic lesions (CIN). The authors found that innate immune pathways in early-stage CeCa were significantly upregulated in response to the infection of human papillomavirus compared with CIN as precursor lesions. The tumors with active immune infiltration showed a more differentiated phenotype, while the ones with increased expression of genes associated with angiogenesis were related to exacerbation of de-differentiation and epithelial-to-mesenchymal transition. As reported, anti-angiogenesis therapy was expected to improve immune evasion and enhance immune infiltration. Shafqat et al. have summarized previous studies that focused on tumor angiogenesis and coagulation exhibiting suppressive effects on the anti-tumor immune response. Both mediate the impacts above through specific mechanisms ultimately leading to the reprogramming of TME. The usage of anti-angiogenic therapies to achieve vascular normalization and ACEi/ARBs to achieve stromal normalization targeting angiogenesis, or anticoagulants targeting coagulation, showed similar results of limiting tumor progression and transforming TME to favor an immune response against tumors. Also, they both demonstrated the efficacy of enhancing immunotherapy while combining with ICIs even though the heterogeneity of tumor types still needs to be considered in conjunction. It is natural to associate angiogenesis with prognosis because of its influence on tumor invasion and progression. Li et al. have developed an advanced machine learning model to evaluate the prognosis and therapy response of breast cancer based on several genes related to vascular mimicry (VM), the performance of which continuously exceeds that of traditional ones. The tumor group received higher VM scores than the normal group. The high-risk group showed a higher level of tumor mutation burden (TMB), higher mutation rate of tumor suppressor genes, and higher level of immune escape, suggesting a poor response to immunotherapy at the same time. Meanwhile, the low-risk group appeared more favorable response to immune checkpoint blockage. Eventually, the authors identified several promising chemotherapy drugs to which VM-related genes were sensitive.

To summarize, the studies above introduce some latest advances in the roles of angiogenesis and immune response in TME of solid tumors and hopefully contribute to the further exploration of immunotherapy enhancers. We would like to acknowledge the invaluable support of all authors, reviewers, and the editorial team during the topic preparation process. We are looking forward to the rapid emergence of significant breakthroughs in the approach of sensitizing immunotherapy through anti-angiogenesis agents.
